# The Cuprizone Mouse Model: A Comparative Study of Cuprizone Formulations from Different Manufacturers

**DOI:** 10.3390/ijms241310564

**Published:** 2023-06-23

**Authors:** Malena Beecken, Louise Baumann, Elise Vankriekelsvenne, Katerina Manzhula, Theresa Greiner, Leo Heinig, Steffen Schauerte, Markus Kipp, Sarah Joost

**Affiliations:** 1 Institute of Anatomy, Rostock University Medical Center, 18057 Rostock, Germany; 2 Institute of Organic Chemistry, RWTH Aachen University, 52074 Aachen, Germany

**Keywords:** Cuprizone mouse model, demyelination, astrogliosis, microgliosis

## Abstract

The Cuprizone mouse model is widely used in studies on de- and remyelination. In the hands of different experimenters, the Cuprizone concentrations that lead to comparable levels of demyelination differ considerably. The reasons for this variability are unknown. In this study, we tested whether different Cuprizone formulations from different vendors and manufacturers influenced Cuprizone-induced histopathological hallmarks. We intoxicated male C57BL/6 mice with six Cuprizone powders that differed in their manufacturer, vendor, and purity. After five weeks, we analyzed the body weight changes over the course of the experiment, as well as the demyelination, astrogliosis, microgliosis and axonal damage by histological LFB-PAS staining and immunohistochemical labelling of PLP, IBA1, GFAP and APP. All Cuprizone formulations induced demyelination, astrogliosis, microgliosis, axonal damage and a moderate drop in body weight at the beginning of the intoxication period. In a cumulative evaluation of all analyses, two Cuprizone formulations performed weaker than the other formulations. In conclusion, all tested formulations did work, but the choice of Cuprizone formulation may have been responsible for the considerable variability in the experimental outcomes.

## 1. Introduction

The Cuprizone mouse model is a well-established model for studying de- and remyelination processes [1,2]. Oral intoxication with Cuprizone leads to myelin degeneration, microgliosis, astrogliosis and axonal damage [3,4,5,6] in various central nervous system regions, such as the corpus callosum, cortex or hippocampal formation [7,8,9]. While microglia and astrocytes are profoundly activated in Cuprizone-induced lesions, the adaptive immune system and invasion of peripheral immune cells play minor roles in the Cuprizone model [10]. The mechanism of Cuprizone-induced demyelination is not entirely understood, as recently reviewed by Zirngibl et al. [11]. Studies have suggested that intoxication with the copper chelator Cuprizone leads to copper deficiency and, therefore, inactivation of copper-dependent respiratory chain enzymes [12]. The resulting changes in mitochondrial homeostasis and metabolic stress are supposed to lead to the apoptosis of mature oligodendrocytes [13,14]. However, since copper supplementation during Cuprizone intoxication does not prevent demyelination, the induction of copper deficiency is likely not the sole mechanism of action of Cuprizone [15,16]. Additionally, microglial and astroglial activation have been described to play significant roles in the induction of oligodendrocyte cell death by the secretion of proinflammatory cytokines [17,18]. It remains unclear whether microglia and astroglia are activated by direct interaction with Cuprizone or by primarily damaged oligodendrocytes. Furthermore, while the most prominent mechanism of Cuprizone-induced oligodendrocyte death is apoptosis [19], the alternative pathways of ferroptosis [20], necroptosis [21] and pyroptosis are likely also of relevance in the Cuprizone model. After Cuprizone withdrawal, proliferating oligodendrocyte precursors differentiate into mature myelinating oligodendrocytes, and endogenous remyelination processes can be studied in the affected regions [22,23]. The Cuprizone model is a valuable tool for studying the consequences of progressive demyelination processes and mechanisms of endogenous remyelination, for example, in the context of secondary progressive multiple sclerosis, a disease of progressive demyelination and axonal degeneration with a largely unknown pathogenesis [24].

Although the Cuprizone model is known as a rather robust model of high reproducibility, many factors such as animal age, weight, strain or chow formulation [2,25,26,27,28] can considerably influence experimental outcomes. An additional potential influencing factor may be the Cuprizone itself. Cuprizone powder is available from various manufacturers in varying degrees of purity, and the influence of these different formulations has never been systematically assessed.

In this study, we tested whether Cuprizone formulations from different manufacturers and in different purities were all equally suited for use in the Cuprizone model with regard to the induction of weight loss, demyelination, microgliosis, astrogliosis and axonal damage in mice.

## 2. Results

The purity levels of all the Cuprizone formulations were tested by means of ^1^H NMR spectroscopy. The results are shown in Table 1 and Figure S1. All the Cuprizone formulations had purity levels above 95%, and in all formulations, minor amounts of cyclohexanone were detected.

**Figure 1:**
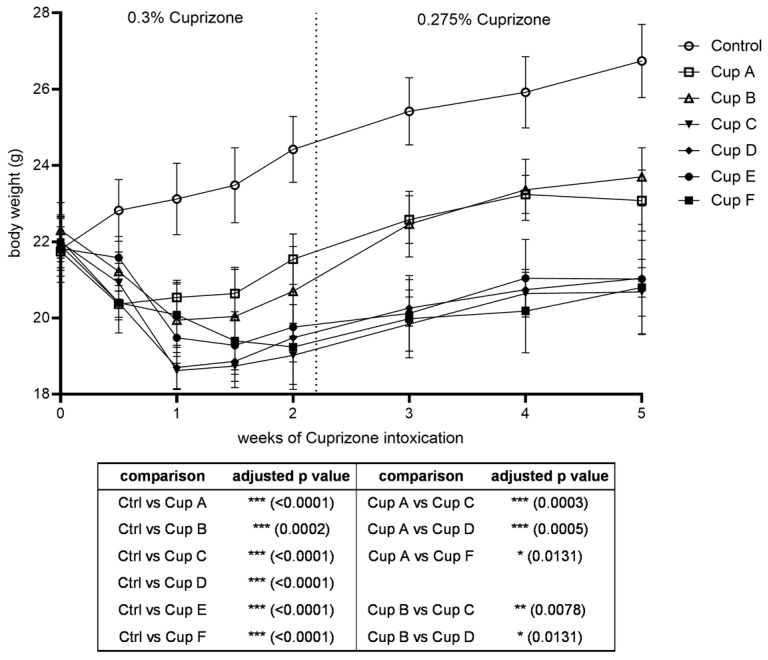
Mouse weights during Cuprizone intoxication with different Cuprizone formulations: the body weights of the mice during the five weeks of Cuprizone intoxication with six different Cuprizone formulations (labelled Cup A–F) and the control animals, with adjusted *p*-values for the significant differences between groups provided below the diagram. Statistical testing was performed using two-way ANOVA for repeated measures with Geisser–Greenhouse correction and Tukey’s multiple comparisons test.

We performed Cuprizone intoxication for five weeks using six different Cuprizone formulations (labelled Cuprizone A–F). The experiment was started with a Cuprizone concentration of 0.3% mixed into ground chow. Animal weights were monitored as a moderate body weight drop is expected at the beginning of Cuprizone intoxication. This weight loss was observed in all groups within the first week of intoxication (Figure 1, body weight in relation to starting weight in Figure S2). To reduce animal stress and prevent severe side effects, the Cuprizone concentrations were decreased to 0.275% on day 16. Afterwards, mouse body weights stabilized in all groups and slightly increased over the rest of the experimental course. Of note, intoxication with Cuprizone A and B led to less severe weight loss than the other Cuprizone formulations throughout the experiment (body weights at the end of the experiment: control, 26.7 ± 1.0 g; Cuprizone A, 23.1 ± 0.8 g; Cuprizone B, 23.7 ± 0.8 g; Cuprizone C, 20.7 ± 0.6 g; Cuprizone D, 21.0 ± 0.5 g; Cuprizone E, 21.0 ± 1.4 g; and Cuprizone F, 20.8 ± 1.2 g).

After five weeks of Cuprizone intoxication, the extent of demyelination, microgliosis, astrogliosis and axonal damage were analyzed in the corpus callosum in two regions of the CNS: in region 265 on the level of the rostral hippocampus, where Cuprizone-induced histopathology is expected to be most severe in the midline of the corpus callosum, and in region 215 at the level of the anterior commissure, where the lateral corpus callosum is mainly affected.

**Figure 2:**
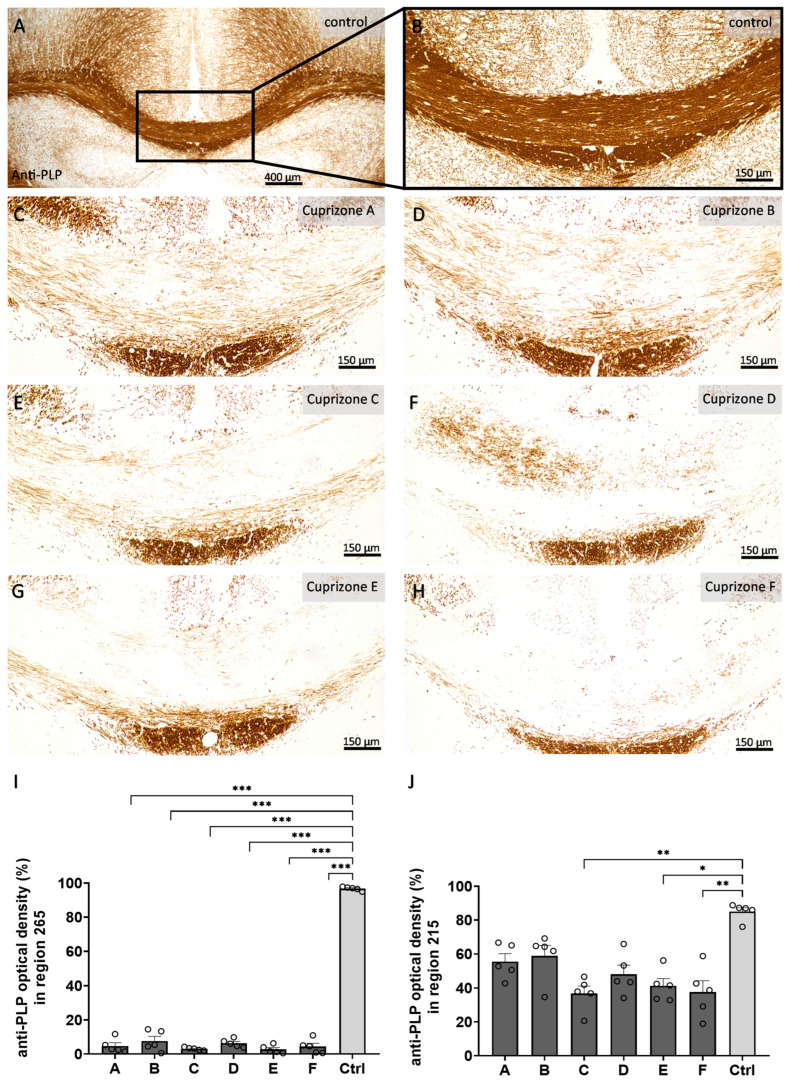
Cuprizone-induced demyelination analyzed by the optical density of the anti-PLP labelling. (**A**–**H**) Representative images of the anti-PLP immunolabelling of the medial corpus callosum in region 265 in control animals (**A**), magnified in (**B**), and in animals intoxicated with six different Cuprizone formulations for five weeks (**C**–**H**). (**I**) Quantification of the optical density of the anti-PLP immunolabelling for region 265 in the medial corpus callosum samples. Statistical testing was performed using ordinary one-way ANOVA with Tukey’s multiple comparisons test. (**J**) Quantification of the optical density of the anti-PLP immunolabelling for region 215 in the lateral corpus callosum samples. Statistical testing was performed using Kruskal–Wallis test with Dunn’s multiple comparisons test. * *p* ≤ 0.05, ** *p* ≤ 0.01, *** *p* ≤ 0.001.

**Figure 3:**
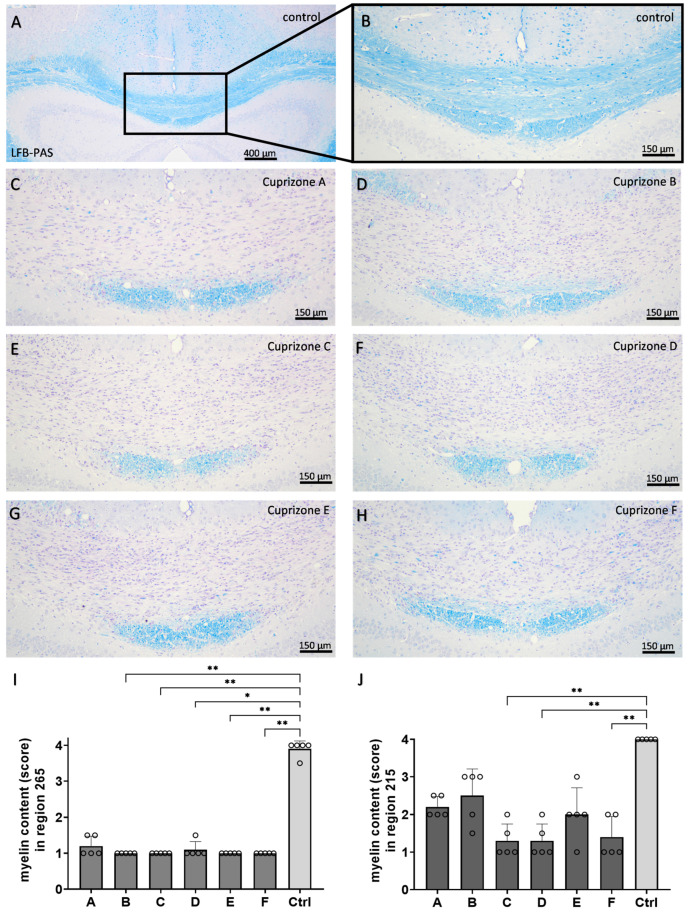
Cuprizone-induced demyelination analyzed by LFB-PAS staining. (**A**–**H**) Representative images of the LFB-PAS staining of the medial corpus callosum in region 265 in the control animals (**A**), magnified in (**B**), and in the animals intoxicated with six different Cuprizone formulations for five weeks (**C**–**H**). (**I**) Quantification of the LFB-PAS staining by scoring for region 265 in the medial corpus callosum. Statistical testing was completed using Kruskal–Wallis tests with Dunn’s multiple comparisons tests. (**J**) Quantification of the LFB-PAS staining by scoring for region 215 in the lateral corpus callosum. Statistical testing was performed using Kruskal–Wallis tests with Dunn’s multiple comparisons test. * *p* ≤ 0.05, ** *p* ≤ 0.01.

Firstly, we analyzed the myelin content in the corpus callosum samples by anti-PLP immunohistochemistry (Figure 2 and Figure S3). In the control animals, the corpus callosum samples were completely myelinated. The optical density of anti-PLP labelling was 96.8 ± 0.5% in the medial corpus callosum for region 265 and 85.0 ± 2.3% in the lateral corpus callosum for region 215. All Cuprizone groups showed nearly complete demyelination of the medial corpus callosum for region 265 (Cuprizone A, 4.8 ± 1.8%; Cuprizone B, 7.6 ± 2.7%; Cuprizone C, 3.1 ± 0.4%; Cuprizone D, 6.4 ± 1.1%; Cuprizone E, 2.7 ± 1.1%; and Cuprizone F, 4.4 ± 1.9%) and moderate demyelination of the lateral corpus callosum for region 215 (Cuprizone A, 55.6 ± 4.5%; Cuprizone B, 58.9 ± 6.2%; Cuprizone C, 36.8 ± 4.4; Cuprizone D, 48.2 ± 5.4%; Cuprizone E, 41.3 ± 4.2%; and Cuprizone F, 37.7 ± 2.3%). These findings were confirmed by histological LFB-PAS staining (Figure 3 and Figure S3). While the myelin contents were not considerably diminished in the control animals (myelin scores of 3.9 ± 0.1 for region 265 and 4.0 ± 0 for region 215), Cuprizone intoxication led to significant decreases in the myelin contents for both regions (region 265: Cuprizone A, 1.2 ± 0.1; Cuprizone B, 1.0 ± 0.0; Cuprizone C, 1.0 ± 0.0; Cuprizone D, 1.1 ± 0.1; Cuprizone E, 1.0 ± 0.0; and Cuprizone F, 1.0 ± 0.0; region 215: Cuprizone A, 2.2 ± 0.1; Cuprizone B, 2.5 ± 0.3; Cuprizone C, 1.3 ± 0.2; Cuprizone D, 1.3 ± 0.2; Cuprizone E, 2.0 ± 0.3; and Cuprizone F, 1.4 ± 0.2).

**Figure 4:**
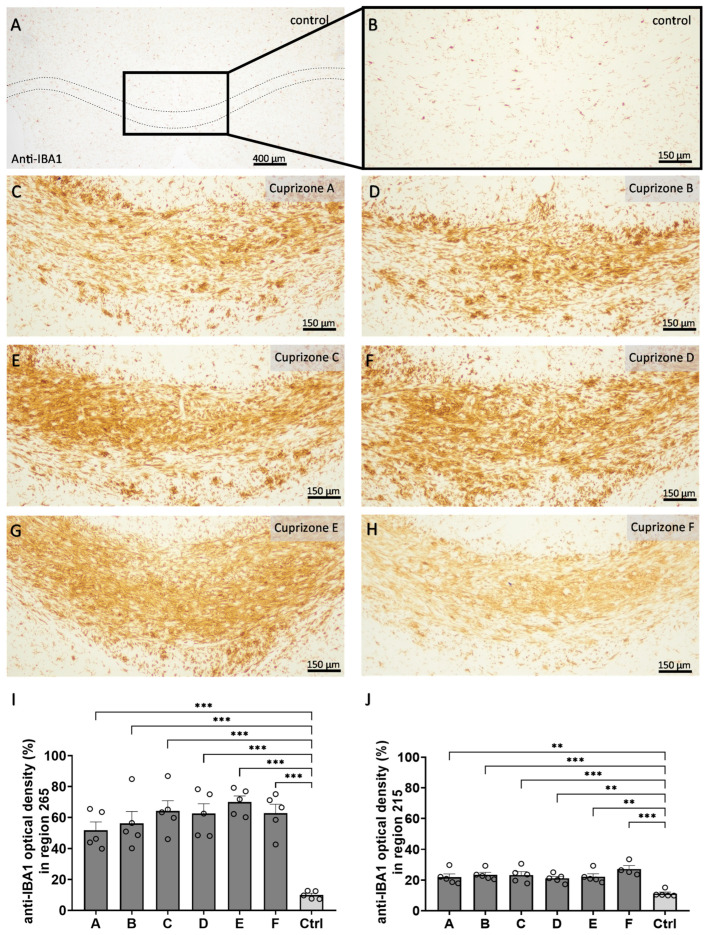
Cuprizone-induced microgliosis analyzed by the optical density of the anti-IBA1 labelling. (**A**–**H**) Representative images of anti-IBA1 immunolabelling of the medial corpus callosum in region 265 in the control animals (dotted line demarcates the corpus callosum in (**A**), magnification in (**B**)). and of the animals intoxicated with six different Cuprizone formulations for five weeks (**C**–**H**). (**I**) Quantification of the optical density of the anti-IBA1 immunolabelling for region 265 in the medial corpus callosum samples. Statistical testing was performed using ordinary one-way ANOVA with Tukey’s multiple comparisons test. (**J**) Quantification of the optical density of the anti-IBA1 immunolabelling for region 215 in the lateral corpus callosum samples. Statistical testing was performed using ordinary one-way ANOVA with Tukey’s multiple comparisons test. ** *p* ≤ 0.01, *** *p* ≤ 0.001.

**Figure 5:**
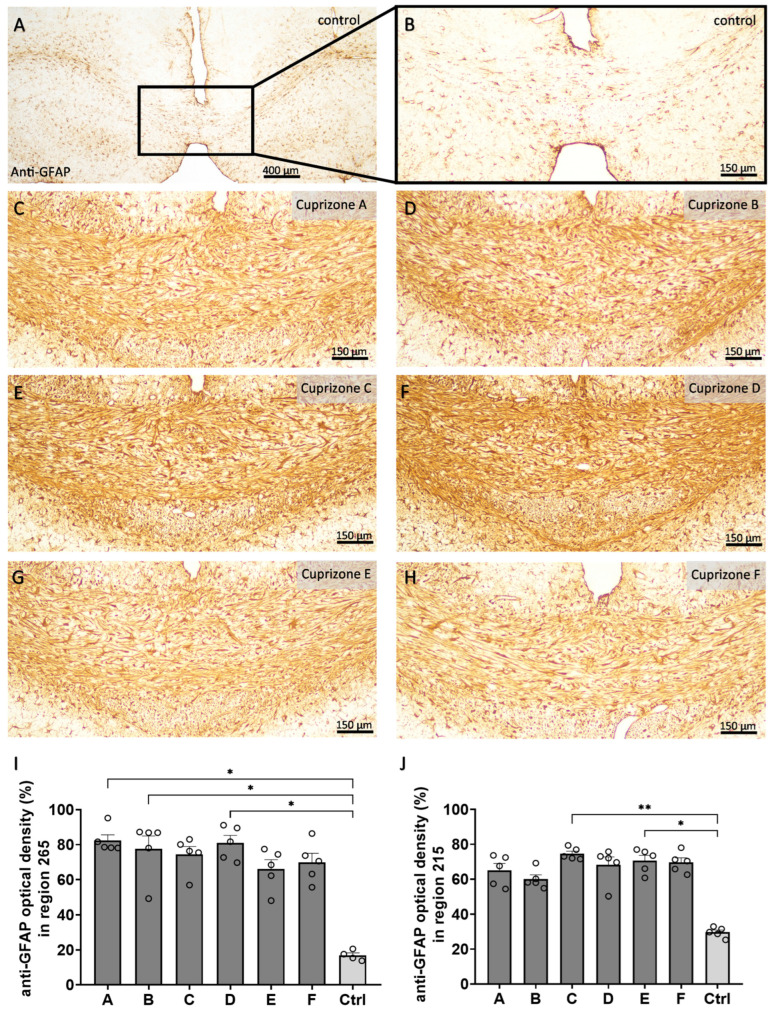
Cuprizone-induced astrogliosis analyzed by the optical density of the anti-GFAP labelling. (**A**–**H**) Representative images of anti-GFAP immunolabelling of the medial corpus in region 265 in the control animals (**A**), magnification in (**B**) and in the animals intoxicated with six different Cuprizone formulations for five weeks (**C**–**H**). (**I**) Quantification of the optical density of the anti-GFAP immunolabelling for region 265 in the medial corpus callosum. Statistical testing was completing using Kruskal–Wallis tests with Dunn’s multiple comparisons tests. (**J**) Quantification of the optical density of the anti-GFAP immunolabelling for region 215 in the lateral corpus callosum samples. Statistical testing was completed using Kruskal–Wallis tests with Dunn’s multiple comparisons tests. * *p* ≤ 0.05, ** *p* ≤ 0.01.

In a second step, we analyzed the extent of microgliosis (Figure 4 and Figure S4) and astrogliosis (Figure 5 and Figure S4) by anti-IBA1 and anti-GFAP immunohistochemistry, respectively. In the control tissues, anti-IBA1-labeled cells were homogenously distributed and displayed thin processes and small cell bodies, resulting in low optical density for the anti-IBA1 labelling (region 265: 10.0 ± 1.1% and region 215: 11.2 ± 1.0%). In all Cuprizone groups, the anti-IBA1-labelled areas had significantly increased, indicating severe reactive microgliosis at the sites of demyelination (region 265: Cuprizone A, 51.9 ± 5.3%; Cuprizone B, 56.2 ± 7.6%; Cuprizone C, 64.3 ± 6.6%; Cuprizone D, 62.5 ± 6.3%; Cuprizone E, 70.1 ± 3.8%; and Cuprizone F, 62.8 ± 1.1%; region 215: Cuprizone A, 21.9 ± 2.1%; Cuprizone B, 23.5 ± 1.5%; Cuprizone C, 23.3 ± 2.2%; Cuprizone D, 21.1 ± 1.3%; Cuprizone E, 22.2 ± 1.9%; and Cuprizone F, 27.2 ± 2.2%). Of note, the extent of microgliosis was more pronounced in the medial corpus callosum in region 265 than in the lateral corpus callosum for region 215. The analysis of anti-GFAP immunohistochemistry revealed similar results. The corpus callosum from the control animals contained anti-GFAP labelled cells of typical astroglial shape, with delicate cell bodies and processes and, therefore, they showed low optical density for the anti-GFAP labelling (region 265: 16.8 ± 1.5% and region 215: 29.7 ± 1.3%). In all Cuprizone groups, the optical density of the anti-GFAP labelling in the corpus callosum increased for both regions, indicating astrogliosis (region 265: Cuprizone A, 82.4 ± 3.3%; Cuprizone B, 77.7 ± 7.3%; Cuprizone C, 74.4 ± 4.6%; Cuprizone D, 81.0 ± 4.3%; Cuprizone E, 66.2 ± 5.2%; and Cuprizone F, 69.9 ± 1.5%; region 215: Cuprizone A, 65.1 ± 3.9%; Cuprizone B, 60.1 ± 2.4%; Cuprizone C, 74.6 ± 1.5%; Cuprizone D, 68.2 ± 4.6%; Cuprizone E, 70.6 ± 3.1%; and Cuprizone F, 69.6 ± 2.6%).

**Figure 6:**
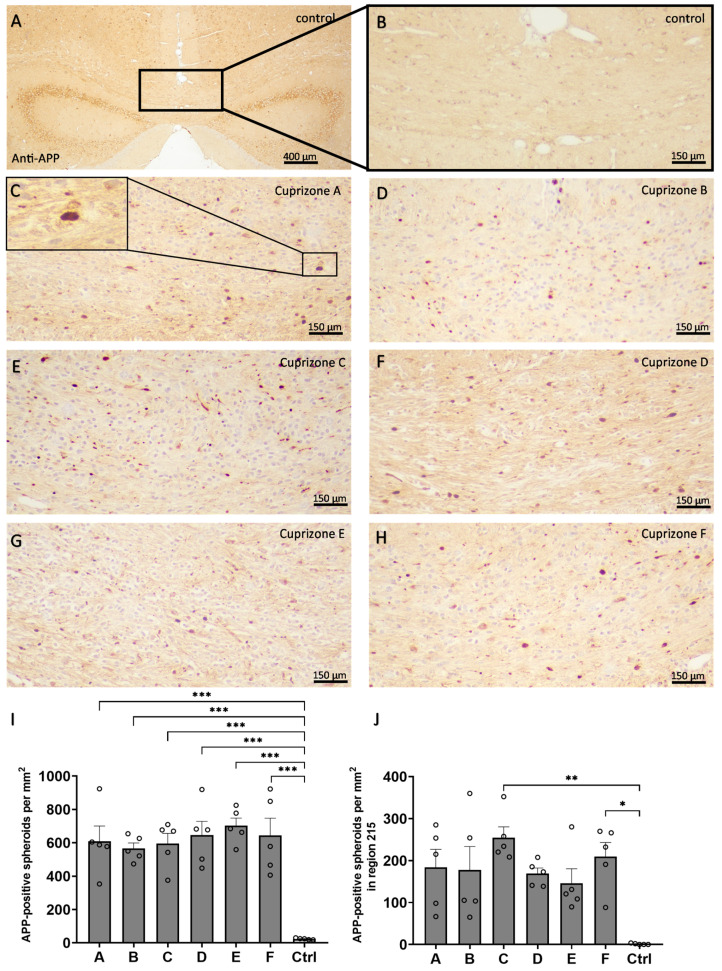
Cuprizone-induced axonal damage analyzed by the optical density of the anti-APP labelling. (**A**–**H**) Representative images of the anti-APP immunolabelling of the medial corpus callosum in region 265 in the control animals (**A**), magnification in (**B**) and in the animals intoxicated with six different Cuprizone formulations for five weeks (**C**–**H**). The magnification in (**C**) shows an APP-positive spheroid. (**I**) Quantification of the density of the anti-APP labelled spheroids for region 265 in the medial corpus callosum. Statistical testing was performed using ordinary one-way ANOVA with Tukey’s multiple comparisons test. (**J**) Quantification of the density of the anti-APP labelled spheroids for region 215 in the lateral corpus callosum. Statistical testing was performed using Kruskal–Wallis test with Dunn’s multiple comparisons test. * *p* ≤ 0.05, ** *p* ≤ 0.01, *** *p* ≤ 0.001.

In a third step, acute axonal damage was assessed by the quantification of APP-positive spheroids resulting from axonal transport interruption (Figure 6). In the medial corpus callosum in region 265, in the control animals, very few APP-positive spheroids were found (23.6 ± 2.2 per mm^2^), while in all the Cuprizone groups, APP-positive spheroids occurred in comparable quantities (Cuprizone A, 609.6 ± 91.1 per mm^2^; Cuprizone B, 565.4 ± 33.4 per mm^2^; Cuprizone C, 595.1 ± 61.8 per mm^2^; Cuprizone D, 646.1 ± 82.8 per mm^2^; Cuprizone E, 702.0 ± 46.4 per mm^2^; and Cuprizone F, 644.5 ± 103.0 per mm^2^). For region 215, the lateral corpus callosum in control animals contained almost no APP-positive spheroids (1.2 ± 0.8 per mm^2^), while in all the Cuprizone groups, APP-positive spheroids were found (Cuprizone A, 183.7 ± 43.1 per mm^2^; Cuprizone B, 177.6 ± 55.9 per mm^2^; Cuprizone C, 254.5 ± 25.8 per mm^2^; Cuprizone D, 169.1 ± 13.2 per mm^2^; Cuprizone E, 146.0 ± 34.3 per mm^2^; and Cuprizone F, 209.4 ± 33.5 per mm^2^).

**Figure 7:**
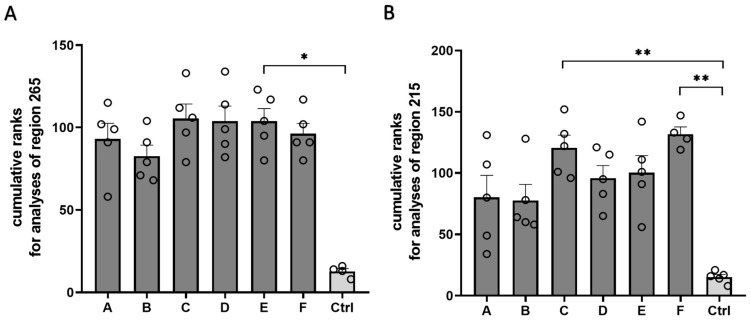
Evaluation of the efficacy of the different Cuprizone formulations by cumulative analysis of all measured parameters. (**A**,**B**) Cumulative ranks for all analyses performed in this study for region 265 (**A**) and region 215 (**B**) for the control animals and for the animals intoxicated with the six different Cuprizone formulations. Statistical testing was performed using Kruskal–Wallis test with Dunn’s multiple comparisons test. * *p* ≤ 0.05, ** *p* ≤ 0.01.

Our histopathological analyses demonstrated that all the Cuprizone formulations induced profound demyelination. As a final step, we combined the results of all analyses to evaluate whether all the Cuprizone formulations worked with a comparable efficacy or if some formulations tended to perform better than others (Figure 7). For this purpose, all animals in the study were ranked for each analysis shown. The weakest reaction towards Cuprizone induction (i.e., the highest optical density of the anti-PLP labelling, the lowest optical density of the anti-GFAP or the anti-IBA1 labelling, the lowest density of the APP-positive spheroids, and the lowest weight loss) was ranked as one, the second-weakest reaction was ranked as two, etc. Ranks for all the analyses were added for each animal, resulting in cumulative ranks. Averaging the cumulative ranks for all analyzed parameters in region 265 resulted in low cumulative ranks for the control animals (12.8 ± 1.7) and high cumulative ranks on a comparable level for all the Cuprizone animals (Cuprizone A, 93.0 ± 9.6; Cuprizone B, 82.6 ± 6.7; Cuprizone C, 105.4 ± 8.9; Cuprizone D, 103.8 ± 7.7; Cuprizone E, 103.8 ± 7.7; and Cuprizone F, 96.2 ± 6.3). Regarding the analyses performed for region 215, the control animals achieved the lowest cumulative ranks (12.2 ± 1.7), while all the Cuprizone groups showed higher cumulative ranks (Cuprizone A, 61.0 ± 13.1; Cuprizone B, 60.0 ± 8.5; Cuprizone C, 94.6 ± 8.4; Cuprizone D, 78.6 ± 10.1; Cuprizone E, 84.6 ± 10.6; and Cuprizone F, 106.3 ± 8.2). Of note, Cuprizones A and B showed trends toward lower cumulative ranks than the other Cuprizone formulations; however, these trends did not reach statistical significance.

## 3. Discussion

All tested Cuprizone formulations induced demyelination, microgliosis, astrogliosis and acute axonal damage in the corpus callosum of mice after five weeks of intoxication. A comparison of the potency of pathology induction revealed that two of the tested Cuprizone formulations tended to induce a weaker pathology than the other four formulations. This tendency was observed for the results for region 215 in the lateral corpus callosum samples. The Cuprizone-induced histopathological changes were less severe in this region compared to the nearly complete demyelination of the medial corpus callosum in region 265. Therefore, subtle differences in Cuprizone efficacy are more likely to be observed in region 215. However, we would like to stress that all formulations worked sufficiently for the successful use of the Cuprizone model under the applied conditions. Still, considerable differences in efficacy were found between different Cuprizone powders which might be an explanation for variations in experimental outcomes concerning the Cuprizone model. Especially Cuprizone A and B performed weaker in our analyses while Cuprizone C and F showed the highest efficacy in the induction of Cuprizone-specific histopathological changes. Remarkably, Cuprizone formulation B performed weakest in our analysis and showed the highest amount of impurities in the spectrometric analysis. However, for the other formulations, measured purities were comparable and therefore do not explain the variance in efficacy.

A crucial factor for the use of the Cuprizone model is the concentration of the Cuprizone used. Cuprizone concentrations above 0.4% have been described to induce severe weight loss, hydrocephalus development and high mortality rates in mice [15]. In the 1990s, Hiremath et al. [29] established a concentration of 0.2% Cuprizone as safe and reliable for the induction of demyelination in C57BL/6 mice. However, many factors such as mouse strain, sex, age and especially weight [25,26,27,30] influence the extent of the Cuprizone-induced demyelination. Therefore, published protocols differ considerably with regard to the Cuprizone concentrations fed to experimental animals, which range from 0.2 to 0.3% [2].

The purpose of this study was to test the suitability of different Cuprizone formulations for use in the Cuprizone model, therefore a robust intoxication strategy that we routinely use for the Cuprizone model was chosen. We began the experiment with a Cuprizone concentration of 0.3%, and we lowered the concentration depending on mouse weight loss. In our experience, this intoxication strategy is more effective than using a fixed concentration in all experiments. Consequent dose reductions in cases of severe weight loss reduces animal stress throughout the experimental course. It also decreases the risk of adverse effects, such as hydrocephalus development, that would interfere with the experimental outcomes. Underdosing that could result in insufficient Cuprizone-induced demyelination is unlikely since initial weight loss will only occur at Cuprizone doses that also induce demyelination. Therefore, we routinely use the observation of body weights as an internal control for Cuprizone efficacy during the experiment. One could argue, however, that the experiment-specific dose adjustments reduce the comparability to other studies. Considering the range of Cuprizone concentrations used in the literature and the varying efficacy of Cuprizone in different facilities, reproducibility of the Cuprizone model is inevitably limited, to a certain extent. Therefore, we prioritized the establishment of individual robust protocols over standardized protocols as long as the relevant factors influencing Cuprizone efficacy remain uncertain.

In regard to the evaluation of the efficacy of the different Cuprizone formulations in this study, the comparably high dose of Cuprizone used may be a limitation as it could mask slight differences in Cuprizone efficacy. If lower Cuprizone concentrations are used throughout an experiment, slight differences in Cuprizone potency may have more pivotal effects on experimental outcomes. For this study, however, we decided to test Cuprizone efficacies with a robust standard intoxication strategy to obtain application-oriented results and recommendations about the choice of Cuprizone formulations.

Providing Cuprizone in pelleted chow is an alternative to mixing it into ground chow. The use of pelleted chow is more convenient; however, changing the Cuprizone dose in response to mouse weights is only possible if different pellet formulations are available. The reliability of Cuprizone intoxication by pelleted chow has been investigated by different groups, leading to contradictory outcomes [28,31]. It is, therefore, likely that the efficacy of Cuprizone-containing pellets is influenced by further unknown factors. Hence, the use of Cuprizone-containing pellets requires thorough establishment in each facility.

## 4. Materials and Methods

### 4.1. Animals

Male 6-week-old C57BL/6 mice (n = 35) were purchased from Janvier Labs, Le Genest-Saint-Isle, France. The mice were maintained at a maximum of five animals per cage with ad libitum food (Ssniff, Soest, Germany, V1534-000) and water. The mice were kept under standard laboratory conditions (12 h light/dark cycle and a controlled temperature of 22 °C ± 2 °C). The cages were changed weekly, and microbiological monitoring was performed according to the Federation of European Laboratory Animal Science Associations recommendations. The animals were allowed to acclimate to the environment for one week before the beginning of the experiments. The Review Board for the Care of Animal Subjects of the district government of Mecklenburg-Western Pomerania approved all experimental procedures (reference number 7221.3-1-001/19).

### 4.2. Cuprizone Intoxication

The Cuprizone powder was weighed with precision scales and mechanically mixed into ground standard rodent chow (Ssniff, Soest, Germany, V1530-000) using a commercially available kitchen machine (WMF, Geißlingen, Germany, Chromargam Kult X, 0416640011) by mixing for 1 min on the highest speed level. Every 15 s, the mixer was tilted by 45° to ensure the complete mixing of the chow and Cuprizone. The chow was provided to the animals in two separate plastic Petri dishes on the bottom of the cage. The animals were provided with fresh chow every day. The mixing of fresh Cuprizone-chow mixture was performed every second day. During Cuprizone intoxication, the animals’ bedding was changed every second day to reduce the dust exposure of the ground chow. The different Cuprizone powders used are listed in Table 2. Special care was taken to prevent the contamination of the different Cuprizone formulations by using individualized containers and cleaning the mixing machine after each use. All Cuprizone powders were delivered within one month prior to the beginning of the experiment and stored at 4 °C.

The experiment was started with a Cuprizone concentration of 0.3% mixed into ground chow. The mice were weighed twice per week during the first two weeks of the experiment to monitor the expected body weight drop induced by the Cuprizone intoxication. As a majority of the mice exhibited a body weight loss of 10% of their starting weight during this time, we reduced the Cuprizone dose to 0.275%. Body weights were measured weekly until the end of the experiment.

### 4.3. NMR Spectroscopy

The purity of the Cuprizone formulations was analysed by ^1^H-NMR spectroscopy in a VNMR 600 spectrometer (Varian Inc., Palo Alto, CA, USA). Samples were dissolved in deuterated chloroform (40 mg/mL, eurisotop). The ^1^H-NMR spectra were recorded at a spectrometer frequency of 600 MHz, and the chemical shift was reported relative to the solvent’s signal.

### 4.4. Histology

The mice were anesthetized with intraperitoneal ketamine (100 mg/kg) and xylazine (10 mg/kg) and then transcardially perfused with 10 mL phosphate-buffered saline (PBS) and 40 mL of 3.7% phosphate-buffered formalin solution. Their brains were exposed within their skulls and post-fixated overnight in 3.7% phosphate-buffered formalin solution. The brains were dissected and embedded in paraffin, and histological sections were cut in a frontal orientation at a 5 µm thickness. According to the mouse brain atlas by Sidman et al. (http://www.hms.harvard.edu/research/brain/atlas.html, accessed on 23 June 2023), region 215 was defined being at the sectional plane of the anterior commissure (Bregma 0.14 mm in the Paxinos mouse atlas) and region 265 was defined as being the rostral part of the hippocampus (Bregma -1.06 mm in the Paxinos mouse atlas).

For the luxol fast blue combined with periodic acid-Schiff (LFB-PAS) staining, rehydrated sections were incubated in the LFB staining solution (0.1% LFB in 96% ethanol) at 56 °C overnight. On the second day, the sections were differentiated with 0.05% and 0.01% lithium carbonate solution until the desired staining intensity (blue staining of the myelinated fiber tracts and minimal bluish staining of the unmyelinated parenchyma) was reached. Afterwards, PAS counterstaining was performed in 0.5% periodic acid solution, Schiff reagent and Mayers Hematoxylin solution, and sections were dehydrated and embedded with Depex.

Two blinded experimenters evaluated the myelination degrees of the regions of interest (lateral corpus callosum in region 215 and medial corpus callosum in region 265) of the LFB-PAS-stained sections with a score of 1 (complete demyelination), 2 (severe demyelination), 3 (slight demyelination) or 4 (complete myelination). The scores of both experimenters were averaged per animal.

### 4.5. Immunohistochemistry

For immunohistochemistry, the paraffin-embedded sections were rehydrated. Heat-induced antigen retrieval was performed by boiling the samples in a microwave oven for 10 min in Tris/EDTA buffer (pH 10) as indicated in Table 3. Unspecific binding was blocked by incubation in blocking solution (5% normal goat serum in PBS) for 1 h at room temperature. The primary antibodies were diluted in blocking solution as indicated in Table 3, and the sections were incubated overnight at 4 °C in a wet chamber. For secondary antibody binding, the sections were incubated with EnVision+ reagent for 1 h at room temperature. Labelling was visualized by incubation with 3,3′-diaminobenzidine (Agilent DAKO, Santa Clara, CA, USA) for 10 min. The sections were then dehydrated and embedded with Depex.

For the analysis, the sections were digitalized using a brightfield microscope (Nikon Eclipse E200LED equipped with a Nikon DS-Fi3 camera). For region 265, the region of interest was defined as the medial corpus callosum with a vertical line dropped through the apex of the cingulum as a lateral boundary. For region 215, the region of interest was defined as the lateral corpus callosum of the left hemisphere with a medial border at the level of the apex of the cingulum. The optical density of immunohistochemical labelling was measured using Fiji (ImageJ 1.53f51, [32]). The regions of interest were manually outlined. Images were converted to binary pictures by applying automated thresholding (using the threshold algorithm IsoData for the anti-IBA1 and anti-PLP in region 215 and the anti-GFAP in region 215, the threshold algorithm Default for the anti-GFAP in region 265 and the threshold algorithm MaxEntropy for the anti-PLP labelling in region 265). The portion of positive pixels within a region of interest were given in percentage. The densities of the APP-positive spheroids were calculated by manual counting of spheroids within the regions of interest.

For a combined overview of all the quantitative results, we performed ranking analyses as follows: For each analysis (body weight loss relative to starting weight; optical density of PLP, IBA1 and GFAP immunoreactivity; and density of APP-positive spheroids), the results of all animals were sorted, and ranks were assigned to each animal for each analysis. High ranks corresponded to strong Cuprizone-induced reactions (i.e., low PLP immunoreactivity, high IBA1 or GFAP immunoreactivity, high body-weight loss and high density of APP-positive spheroids). The rankings were summed up per animal, and then cumulative ranks were averaged for each experimental group. The analysis was performed for both region 265 and region 215. Two animals were excluded from the analysis because no results was obtained from one of their immunohistochemical labellings, and therefore, ranks from 1 to 34 were assigned for each analysis.

### 4.6. Statistical Analysis

All the data are provided as arithmetic means ± standard error of the means. Statistical testing was performed using Prism 8.0.2 (GraphPad Software Inc., San Diego, CA, USA) with confidence intervals of 0.05. A Shapiro–Wilk test was applied to test for normality distribution. Ordinary one-way ANOVA with Tukey’s multiple comparisons test was applied for normally distributed data. Kruskal–Wallis test with Dunn’s multiple comparisons test was applied for non-normally distributed data and parametric data. Two-way ANOVA for repeated measures with Geisser–Greenhouse correction and Tukey’s multiple comparisons test were applied to compare body weights over multiple time points. For multiple comparisons, main column means were compared. The applied statistical tests are provided in the respective figure legends, and *p*-values of ≤0.05 were considered to be statistically significant. The following symbols were used to indicate the level of significance: * *p* ≤ 0.05, ** *p* ≤ 0.01 and *** *p* ≤ 0.001. No outliers were excluded from the analyses.

## 5. Conclusions

In summary, the tested Cuprizone powders from various manufacturers all induced demyelination, microgliosis, astrocytosis and axonal damage; however, the degree of damage slightly varied. Differences in the Cuprizone formulations by different manufacturers may indeed account for minor variations in the Cuprizone efficacy levels in different facilities. Nonetheless, additional unknown factors are highly probable to influence the experimental outcomes of the Cuprizone model.

## Figures and Tables

**Table 1:** Cuprizone purity level and main impurities assessed by ^1^H NMR spectroscopy.

	Purity (wt%)	Impurities
Cuprizone A	ca. 98%	cyclohexanone (<1%), ethanol (<1%)
Cuprizone B	>95%	cyclohexanone (ca. 3%), non-identified aliphatic compounds (<2%)
Cuprizone C	ca. 98%	cyclohexanone (<1%), ethanol (<1%)
Cuprizone D	ca. 98%	cyclohexanone (<1%), ethanol (<1%)
Cuprizone E	ca. 98%	cyclohexanone (<1%), N,N-dimethylformamide (<1%)
Cuprizone F	ca. 98%	cyclohexanone (<1%), ethanol (<1%)

**Table 2:** Cuprizone formulations used in this study.

	Vendor	Catalogue Number	Purity ^1^
Cuprizone A	Sigma Aldrich (St. Louis, MO, USA)/ Merck (Rahway, NJ, USA)	C9012-25G	95%
Cuprizone B	Sigma Aldrich (St. Louis, MO, USA)/ Merck (Rahway, NJ, USA)	14690-25G	99%
Cuprizone C	Sigma Aldrich (St. Louis, MO, USA)/ Merck (Rahway, NJ, USA)	1.01841.0025	99%
Cuprizone D	Thermo Fisher (Waltham, MA, USA)	A10628	98%
Cuprizone E	TCI (Fremont, CA, USA)	B0476	98%
Cuprizone F	Glentham (Corsham, UK)	GT6965	98%

^1^ as stated by the vendor.

**Table 3:** Antibodies for immunohistochemistry.

Labelling	Antigen Retrieval	Primary Antibody: Catalogue Number, RRID and Dilution	Secondary Antibody
anti-PLP (myelin proteolipid protein)	no retrieval	Bio-Rad, MCA839G, AB_2237198 and 1:5000	EnVision anti-mouse (Dako, K4001)
anti-IBA1 (ionized calcium-binding adaptor molecule 1)	Tris/EDTA	Wako, 019-19741, AB_839504 and 1:5000	EnVision anti-rabbit (Dako, K4003)
anti-GFAP (glial fibrillary acidic protein)	Tris/EDTA	Abcam, ab68428, AB_1209224 and 1:250	EnVision anti-rabbit (Dako, K4003)
anti-APP (amyloid precursor protein)	Tris/EDTA	Millipore, MAB348, AB_94882 and 1:5000	EnVision anti-mouse (Dako, K4001)

## Data Availability

The data are contained within the article or the supplementary materials.

## Supplementary Materials

The following supporting information can be downloaded at: https://www.mdpi.com/article/10.3390/ijms241310564/s1.

## References

[1] Zhan J., Mann T., Joost S., Behrangi N., Frank M., Kipp M. (2020). The Cuprizone Model: Dos and Do Nots. Cells.

[2] Kipp M., Clarner T., Dang J., Copray S., Beyer C. (2009). The cuprizone animal model: New insights into an old story. Acta Neuropathol..

[3] Skripuletz T., Hackstette D., Bauer K., Gudi V., Pul R., Voss E., Berger K., Kipp M., Baumgartner W., Stangel M. (2013). Astrocytes regulate myelin clearance through recruitment of microglia during cuprizone-induced demyelination. Brain.

[4] Höflich K.M., Beyer C., Clarner T., Schmitz C., Nyamoya S., Kipp M., Hochstrasser T. (2016). Acute axonal damage in three different murine models of multiple sclerosis: A comparative approach. Brain Res..

[5] Gudi V., Gingele S., Skripuletz T., Stangel M. (2014). Glial response during cuprizone-induced de- and remyelination in the CNS: Lessons learned. Front. Cell. Neurosci..

[6] Goldberg J., Clarner T., Beyer C., Kipp M. (2015). Anatomical Distribution of Cuprizone-Induced Lesions in C57BL6 Mice. J. Mol. Neurosci..

[7] Schmidt T., Awad H., Slowik A., Beyer C., Kipp M., Clarner T. (2013). Regional heterogeneity of cuprizone-induced demyelination: Topographical aspects of the midline of the corpus callosum. J. Mol. Neurosci..

[8] Koutsoudaki P.N., Skripuletz T., Gudi V., Moharregh-Khiabani D., Hildebrandt H., Trebst C., Stangel M. (2009). Demyelination of the hippocampus is prominent in the cuprizone model. Neurosci. Lett..

[9] Skripuletz T., Lindner M., Kotsiari A., Garde N., Fokuhl J., Linsmeier F., Trebst C., Stangel M. (2008). Cortical Demyelination Is Prominent in the Murine Cuprizone Model and Is Strain-Dependent. Am. J. Pathol..

[10] Kaddatz H., Joost S., Nedelcu J., Chrzanowski U., Schmitz C., Gingele S., Gudi V., Stangel M., Zhan J., Santrau E. (2021). Cuprizone-induced demyelination triggers a CD8-pronounced T cell recruitment. Glia.

[11] Zirngibl M., Assinck P., Sizov A., Caprariello A.V., Plemel J.R. (2022). Oligodendrocyte death and myelin loss in the cuprizone model: An updated overview of the intrinsic and extrinsic causes of cuprizone demyelination. Mol. Neurodegener..

[12] Acs P., Selak M.A., Komoly S., Kalman B. (2013). Distribution of oligodendrocyte loss and mitochondrial toxicity in the cuprizone-induced experimental demyelination model. J. Neuroimmunol..

[13] Hashem M., Shafqat Q., Wu Y., Rho J.M., Dunn J.F. (2022). Abnormal oxidative metabolism in the cuprizone mouse model of demyelination: An in vivo NIRS-MRI study. Neuroimage.

[14] Praet J., Guglielmetti C., Berneman Z., van der Linden A., Ponsaerts P. (2014). Cellular and molecular neuropathology of the cuprizone mouse model: Clinical relevance for multiple sclerosis. Neurosci. Biobehav. Rev..

[15] Carlton W.W. (1967). Studies on the induction of hydrocephalus and spongy degeneration by cuprizone feeding and attempts to antidote the toxicity. Life Sci..

[16] Tandler B., Hoppel C.L. (1975). The Failure of Supplemental Dietary Copper to Prevent Cuprizone-Induced Alterations in Mouse Hepatocytes. Beiträge Pathol..

[17] Pasquini L.A., Calatayud C.A., Bertone Uña A.L., Millet V., Pasquini J.M., Soto E.F. (2007). The Neurotoxic Effect of Cuprizone on Oligodendrocytes Depends on the Presence of Pro-inflammatory Cytokines Secreted by Microglia. Neurochem. Res..

[18] Marzan D.E., Brügger-Verdon V., West B.L., Liddelow S., Samanta J., Salzer J.L. (2021). Activated microglia drive demyelination via CSF1R signaling. Glia.

[19] Hesse A., Wagner M., Held J., Brück W., Salinas-Riester G., Hao Z., Waisman A., Kuhlmann T. (2010). In toxic demyelination oligodendroglial cell death occurs early and is FAS independent. Neurobiol. Dis..

[20] Jhelum P., Santos-Nogueira E., Teo W., Haumont A., Lenoël I., Stys P.K., David S. (2020). Ferroptosis Mediates Cuprizone-Induced Loss of Oligodendrocytes and Demyelination. J. Neurosci..

[21] Ofengeim D., Ito Y., Najafov A., Zhang Y., Shan B., DeWitt J.P., Ye J., Zhang X., Chang A., Vakifahmetoglu-Norberg H. (2015). Activation of Necroptosis in Multiple Sclerosis. Cell Rep..

[22] Johnson E.S., Ludwin S.K. (1981). The demonstration of recurrent demyelination and remyelination of axons in the central nervous system. Acta Neuropathol..

[23] Islam M.S., Tatsumi K., Okuda H., Shiosaka S., Wanaka A. (2009). Olig2-expressing progenitor cells preferentially differentiate into oligodendrocytes in cuprizone-induced demyelinated lesions. Neurochem. Int..

[24] Filippi M., Bar-Or A., Piehl F., Preziosa P., Solari A., Vukusic S., Rocca M.A. (2018). Multiple sclerosis. Nat. Rev. Dis. Prim..

[25] Taylor L.C., Gilmore W., Matsushima G.K. (2009). SJL mice exposed to cuprizone intoxication reveal strain and gender pattern differences in demyelination. Brain Pathol..

[26] Irvine K.-A., Blakemore W.F. (2006). Age increases axon loss associated with primary demyelination in cuprizone-induced demyelination in C57BL/6 mice. J. Neuroimmunol..

[27] Paton K.F., Hong S., Biggerstaff A., Kivell B.M. (2022). Sex Differences in the Behavioural Aspects of the Cuprizone-Induced Demyelination Model in Mice. Brain Sci..

[28] Toomey L.M., Papini M., Lins B., Wright A.J., Warnock A., McGonigle T., Hellewell S.C., Bartlett C.A., Anyaegbu C., Fitzgerald M. (2021). Cuprizone feed formulation influences the extent of demyelinating disease pathology. Sci. Rep..

[29] Hiremath M.M., Saito Y., Knapp G.W., Ting J.P., Suzuki K., Matsushima G.K. (1998). Microglial/macrophage accumulation during cuprizone-induced demyelination in C57BL/6 mice. J. Neuroimmunol..

[30] Gingele S., Henkel F., Heckers S., Moellenkamp T.M., Hümmert M.W., Skripuletz T., Stangel M., Gudi V. (2020). Delayed Demyelination and Impaired Remyelination in Aged Mice in the Cuprizone Model. Cells.

[31] Hochstrasser T., Exner G.L., Nyamoya S., Schmitz C., Kipp M. (2017). Cuprizone-Containing Pellets Are Less Potent to Induce Consistent Demyelination in the Corpus Callosum of C57BL/6 Mice. J. Mol. Neurosci..

[32] Schindelin J., Arganda-Carreras I., Frise E., Kaynig V., Longair M., Pietzsch T., Preibisch S., Rueden C., Saalfeld S., Schmid B. (2012). Fiji: An open-source platform for biological-image analysis. Nat. Methods.

