# Supplementary figures and images for: The Cuprizone Mouse Model: A Comparative Study of Cuprizone Formulations from Different Manufacturers
Source: Int J Mol Sci. 2023 Jun 23;24(13):10564. doi: 10.3390/ijms241310564 (PMC10341492; doi:10.3390/ijms241310564)

## Cuprizone A

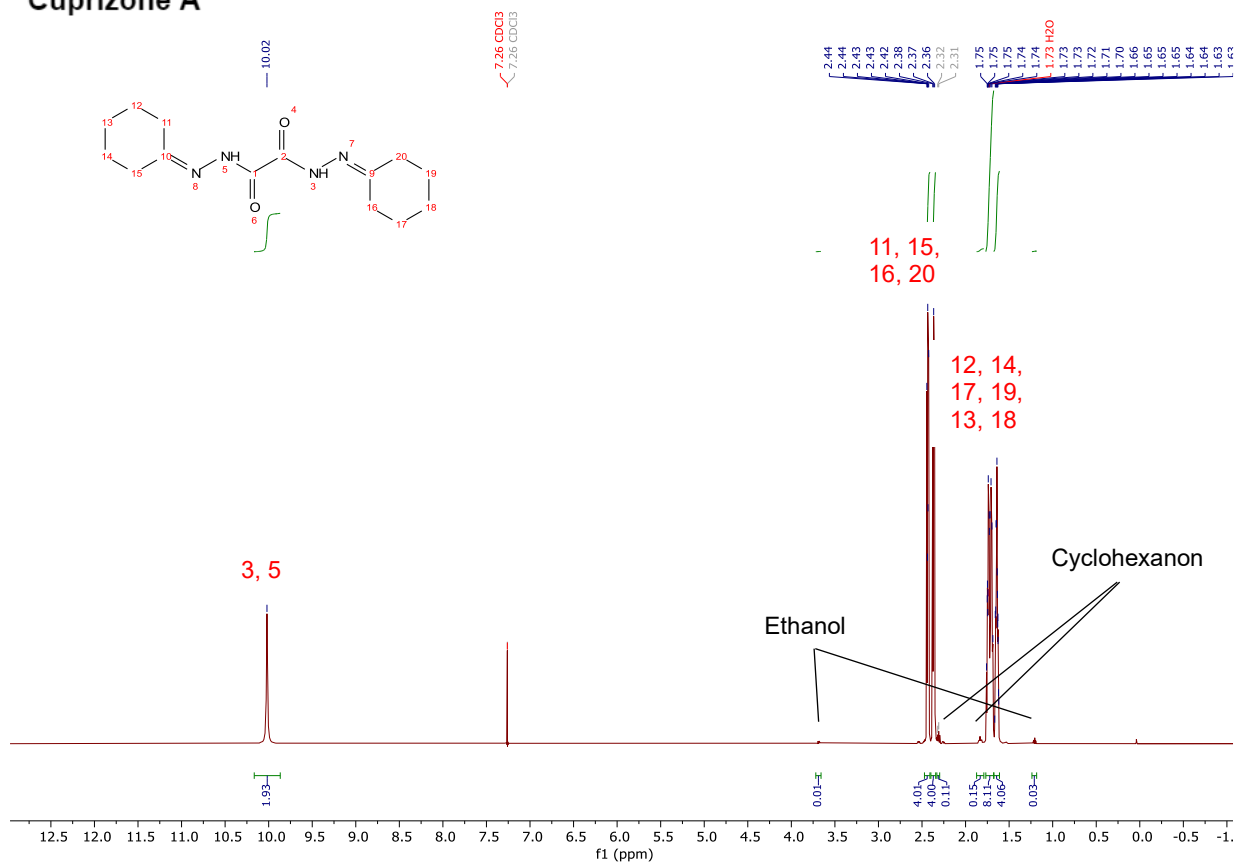

## Cuprizone B

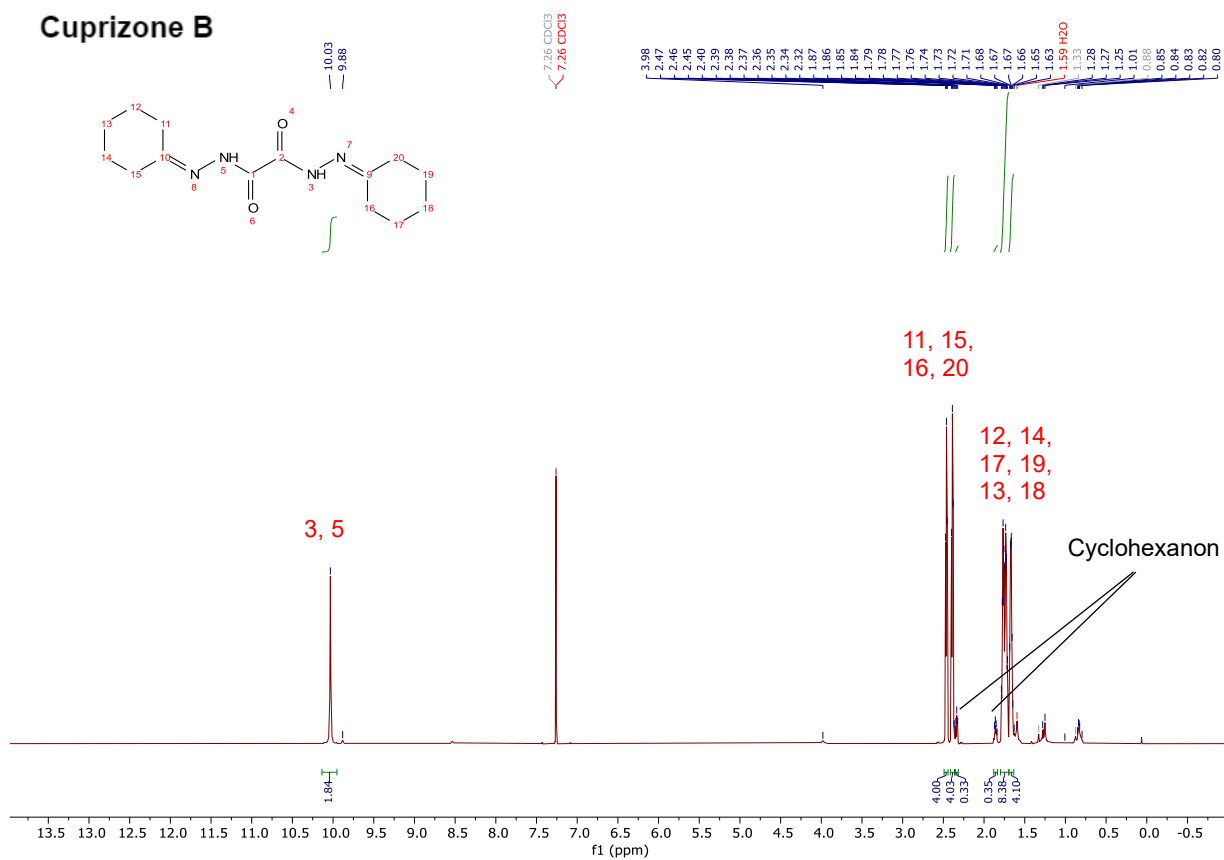

## Cuprizone C

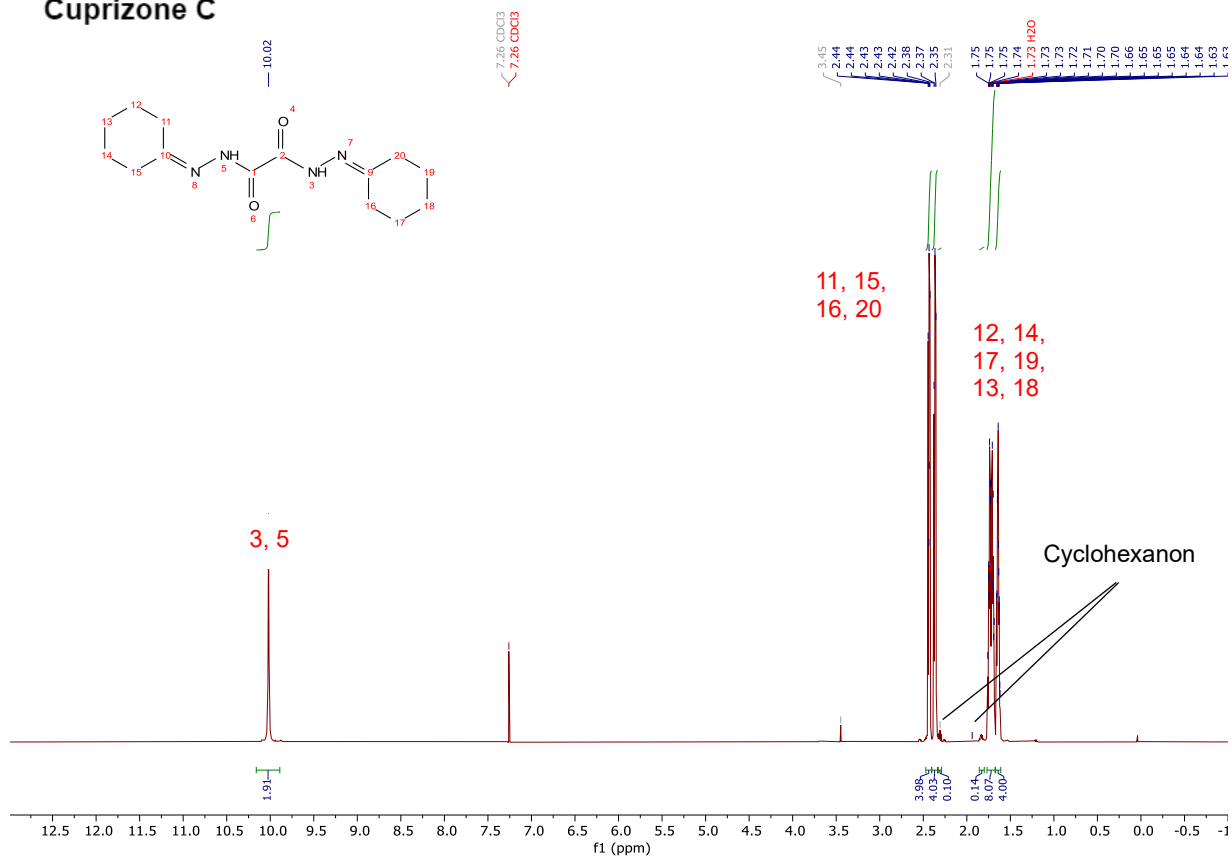

## Cuprizone D

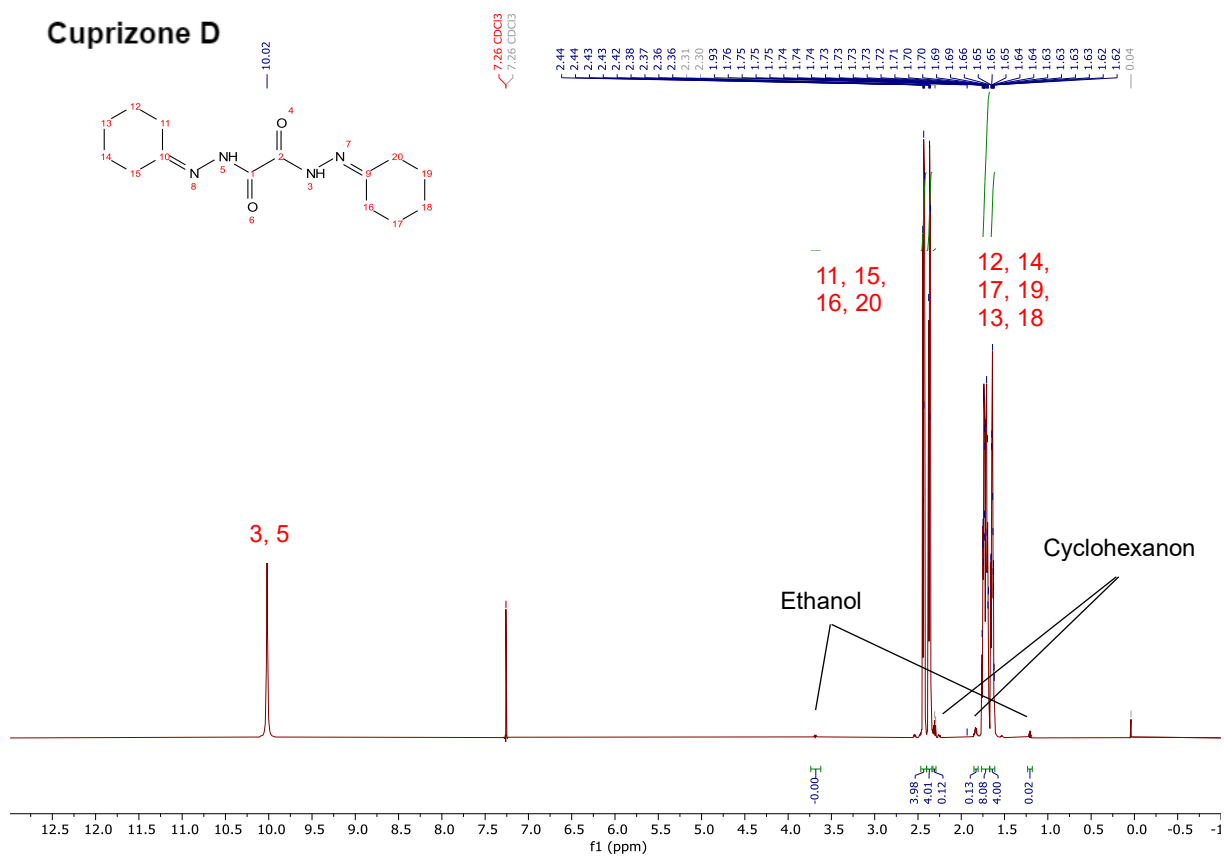

## Cuprizone E

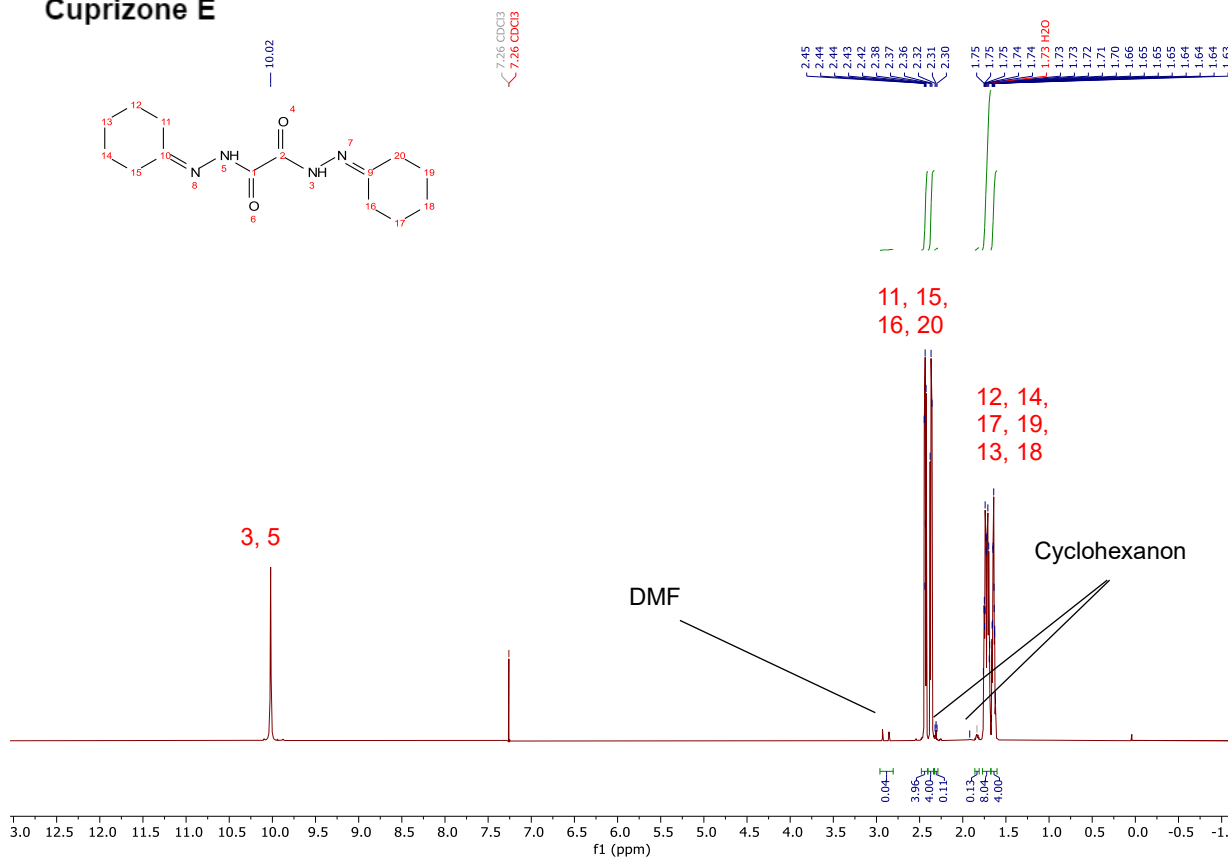

## Cuprizone F

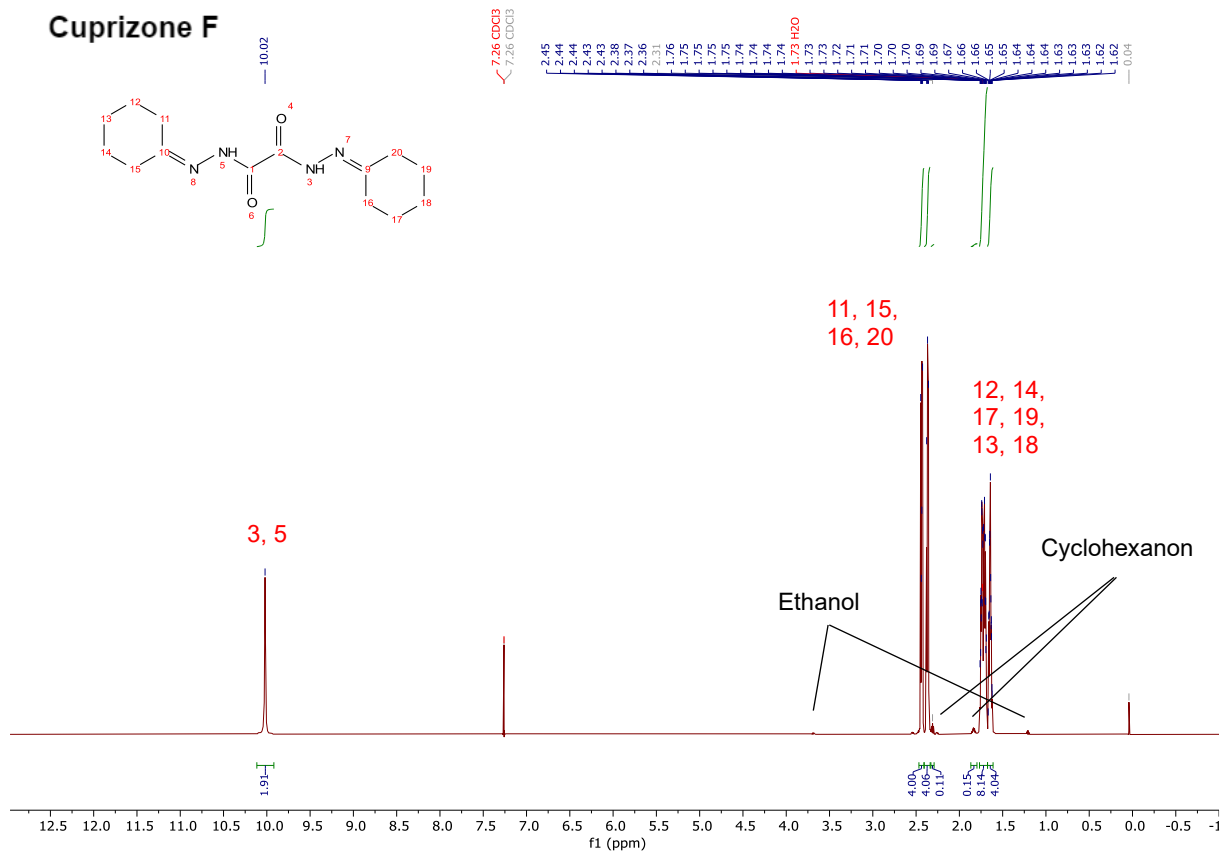

Supplement: Supplementary file 1 [file ijms-24-10564-s001.zip › Supplementary material/Figure S1.pdf]

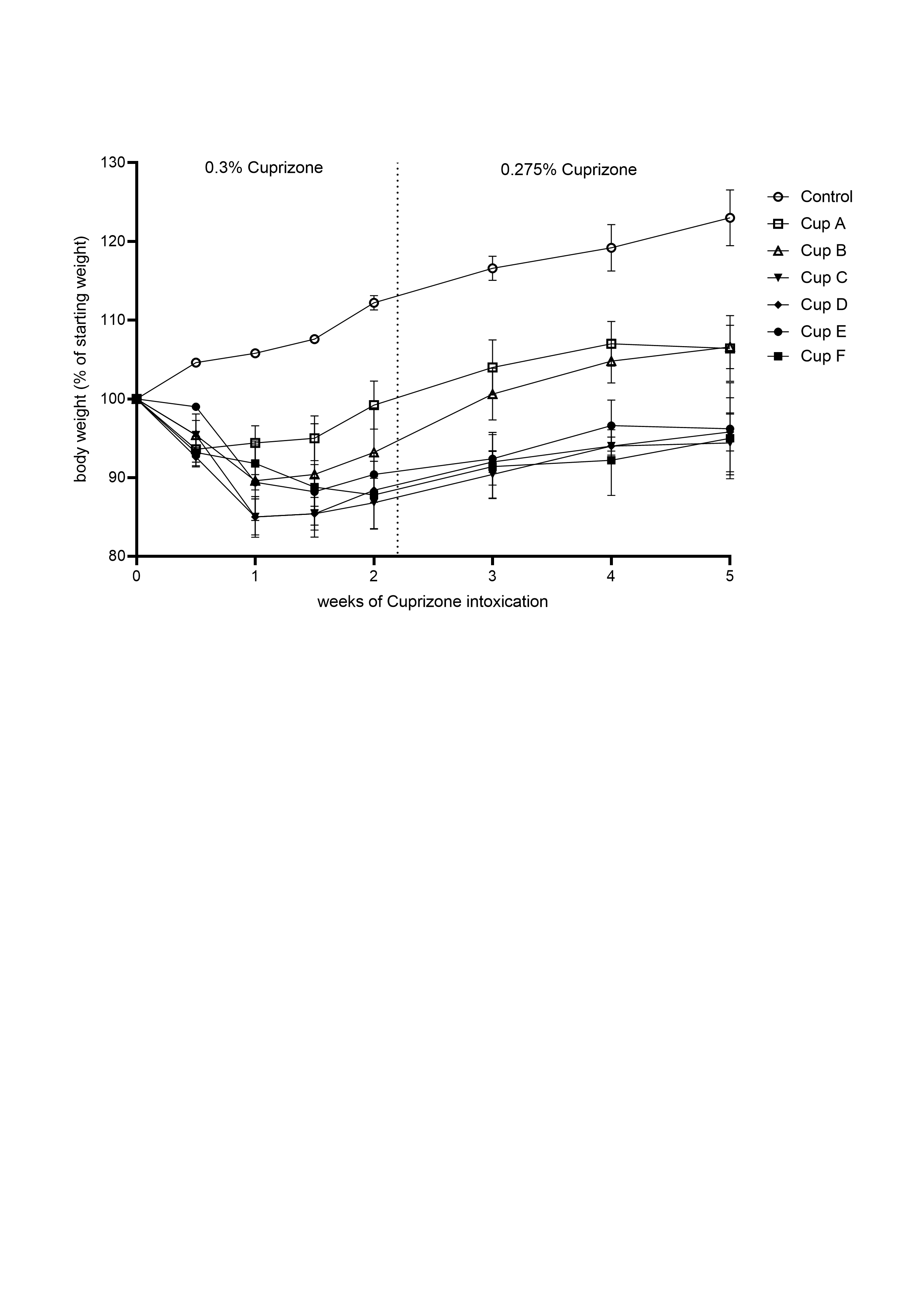

Supplement: Supplementary file 1 [file ijms-24-10564-s001.zip › Supplementary material/Figure S2.tif]

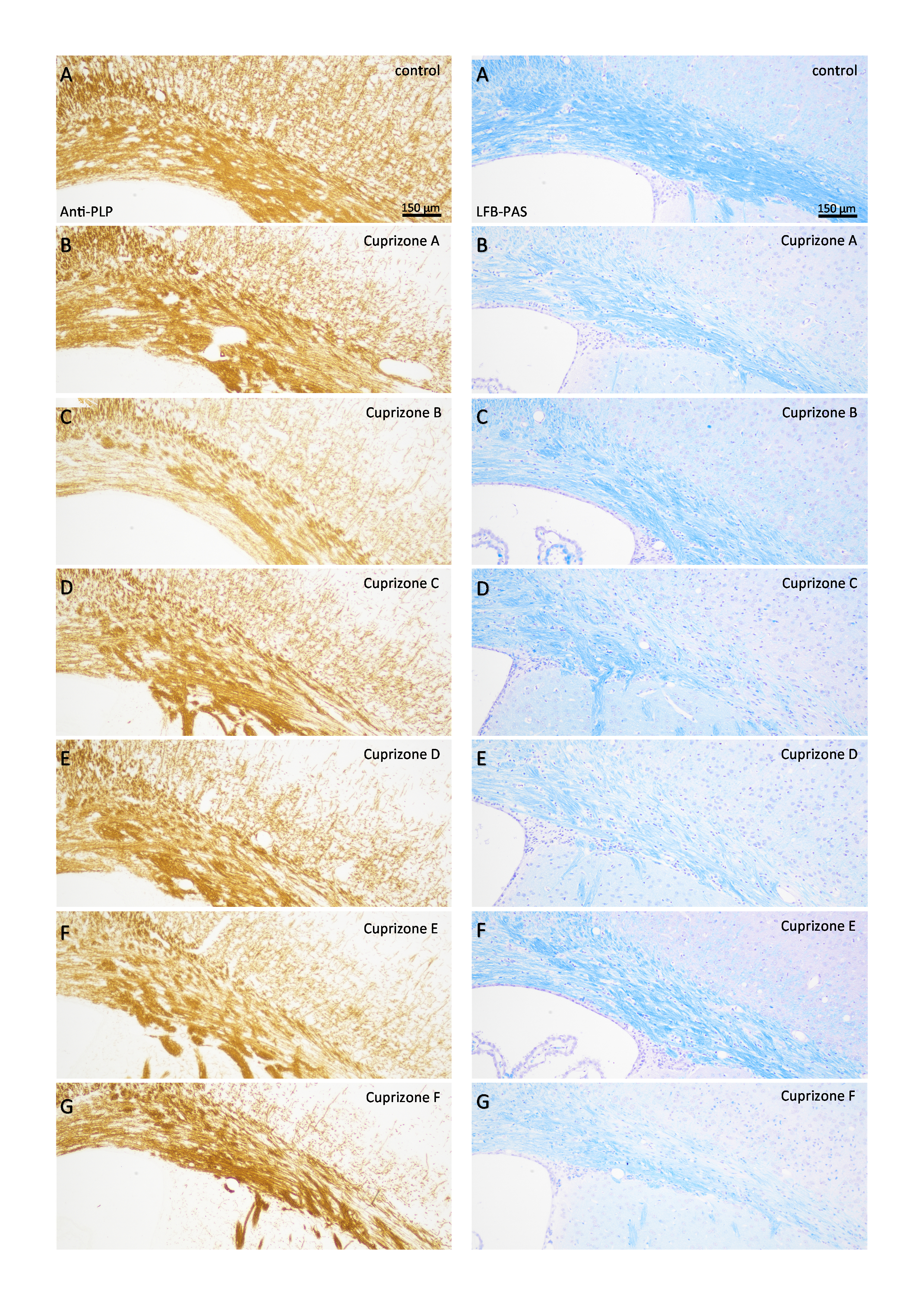

Supplement: Supplementary file 1 [file ijms-24-10564-s001.zip › Supplementary material/Figure S3.tif]

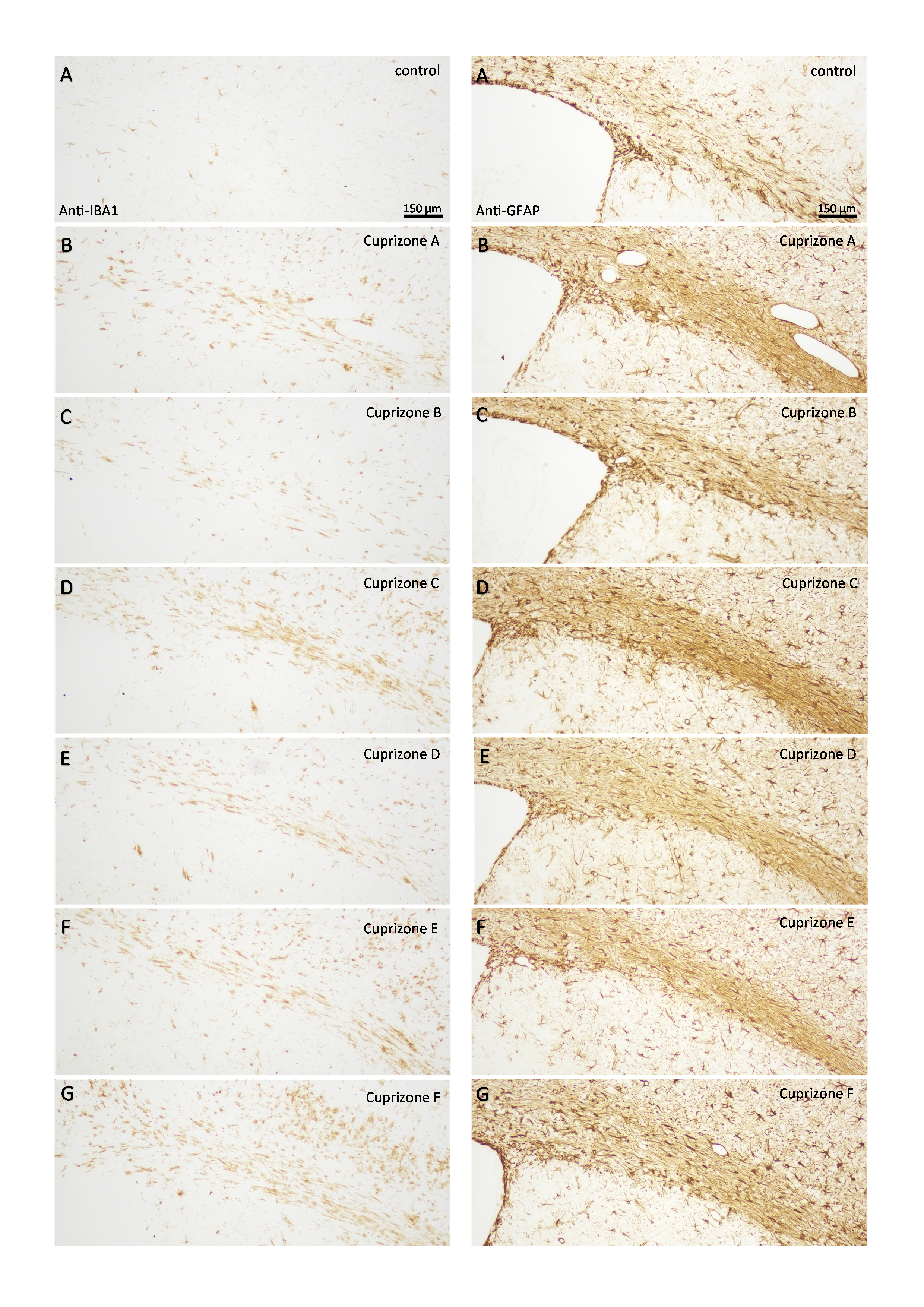

Supplement: Supplementary file 1 [file ijms-24-10564-s001.zip › Supplementary material/Figure S4.tif]
